# The resilience of parents and carers who administer medicines to children at home: a qualitative systematic review protocol

**DOI:** 10.1186/s13643-024-02724-9

**Published:** 2025-01-23

**Authors:** Stephen Morris, Simon Pini, Beth Fylan, Franki Wilson, Helen Faulkner, David P. Alldred

**Affiliations:** 1 School of Healthcare, University of Leeds, Worsley Building, Leeds, LS2 9JT UK; 2 Faculty of Medicine and Health, University of Leeds, Worsley Building, Leeds, LS2 9JT UK; 3 School of Pharmacy and Medical Sciences, Faculty of Life Sciences, University of Bradford, Bradford, BD7 1DP UK

**Keywords:** Systematic review protocol, Systematic review, Qualitative synthesis, Framework synthesis, Medication errors, Administration errors, Pediatrics, Medication administration, Domiciliary, Parents, Guardians, Caregivers

## Abstract

**Background:**

Parents and carers are increasingly expected to administer prescribed medicines to their children at home. However, parents and carers are not always able to administer medicines as directed by the prescriber and ultimately must rely on their own judgment to administer medicines safely. This process is often unseen but may contain important learning for professionals, academics, and wider society. Studying safety in everyday healthcare work presents researchers with many challenges. However, recent developments in our understanding of resilience and how it manifests within healthcare can provide an effective framework for enquiry. The aim of this review is to use resilience theory to explore parents’ and carers’ experiences when administering medicines to children at home.

**Methods:**

This systematic review will follow the framework synthesis method. An iterative search strategy, using a scoping search of the major databases (Embase, PyscINFO, CINAHL, Cochrane and PubMed) will be used. The three main search terms are parents and carers, administration of medicines, and the home environment. Included studies will contain qualitative data and investigate the experiences of parents or carers who administer prescribed medicines to children at home. Relevant studies will be quality assessed using the Joanna Briggs Institute critical appraisal checklist for qualitative research.

Framework synthesis will be completed by following five stages: familiarisation, thematic framework identification, indexing, charting, mapping, and interpretation. The findings identified in the data extraction phase will be indexed and charted according to the three elements of Moments of Resilience theory.

**Discussion:**

This protocol describes a novel method to address an important patient safety issue. A strength of this review will be not only to identify, describe and collate existing studies, but also to learn about the application of resilience theory to a medication safety topic. The knowledge generated from this will inform intervention development to improve the support for families to administer medicines safely at home.

**Systematic review registration:**

This review has been registered on the International Prospective Register of Systematic Reviews database (PROSPERO) #487154.

## Background

Medicines are a cornerstone of modern healthcare. They are the leading patient-level intervention used by the NHS in England which is a testament to their success at treating illness and disease [1]. Despite the benefits of using medicines within an evidence-based approach, there will always be the possibility for medicines to cause harm. In fact, medicines are one of the leading causes of preventable harm within healthcare [2].

There are many ways in which a medicine can cause harm. Unfortunately, some types of harm are unavoidable. For example, an allergic reaction to a penicillin antibiotic in someone with no prior history of allergies. However, there is also harm which could be considered avoidable, for example, harm caused by the incorrect use of medicines, referred to as medication errors [3].

A medication error is defined as a preventable event involving a medicine that leads to or has the potential to, harm a patient [4]. A further sub-category of medication error is an administration error, this occurs when there is a discrepancy between the medicine received by the patient and the medicine therapy intended by the prescriber [5]. For example, an overdose is a type of administration error, which is when more than the intended dose is given [6, 7].

Families are increasingly expected to accept responsibility for and deliver, a variety of healthcare tasks at home [8, 9]. For many children, they will rely on a family member or carer to administer medicine to them on their behalf. When medicines are used in this context, the risk of administration errors is well documented [6, 7, 10–12], with factors such as age [6, 7, 11] and the number of medicines prescribed shown to increase risk [12].

To date, research in this area has primarily used quantitative methods to measure the prevalence of administration errors that occur at home. These studies suggest that parents and carers are able to avoid administration errors with varying degrees of success. It is estimated that administration error rates range from 1.9 to 33% of all medicines given at home [13]. The wide range observed is indicative of the complexity of the task under investigation and the challenges associated with conducting research in this area.

The complexity of medicine administration at home means that there is an almost endless variety of scenarios and methods used to achieve the aim of administering the medicine. Given that the definition of administration error is binary (i.e. an error either occurs or does not), this creates difficulty for researchers who are forced to determine what does or does not meet this definition. Further variation in estimates may also result from the relative weaknesses of the quantitative methods used. For example, by using observations of administration in an outpatient clinic as a surrogate for the administration of medicines at home [12].

Qualitative studies are beginning to explore the phenomenon of parent and carer administration of medicines to children at home in greater depth [14–17]. This small group of studies demonstrates a variety of methods used to explore this topic. For example, data collection has been conducted using face-to-face interviews [14, 15], focus groups [14], diaries [15], and online message boards [16, 17]. Data analysis has primarily been thematic analysis and there is some coherence in the emerging themes. For example, the proactive role of parents in organizing medication administration [14–16] and the willingness of parents to share experiences with one another [14, 16].

There are two qualitative syntheses of these studies, and both used thematic synthesis. One had a focus on health literacy [18] and the other a focus on non-adherence [19]. There has been little advancement of qualitative research to inform intervention development, or other applications to improve patient safety. This will require an approach that is inclusive of both the complexity of the administration of medicines to children at home and which recognises the important role parents and carers undertake, to ensure medicines are safely administered [20]. This is supported by the view that studying what goes right, as well as what goes wrong, is an important approach to improving safety [21].

Resilient Healthcare can provide this approach. Resilient Healthcare is defined as the capacity to adapt to challenges and changes at different system levels in order to maintain high-quality care [22]. Resilience originated as a concept from engineering and can be further described as understanding how individuals, organisations, communities, and systems are able to sustain everyday operations under anticipated and unanticipated conditions [23]. Resilient Healthcare is a developing field of enquiry and Resilient Healthcare theory has started to be applied to deliver meaningful improvements to patient safety [24].

A useful aspect of studying resilience is that it acknowledges that despite perceived disruptions and failures, these events may lead to adaptations and improvements [25]. These disruptions and adaptations could potentially provide one reason as to why, according to quantitative studies, so many medicines are not administered by parents and carers as prescribed by professionals.

**Fig. 1:**
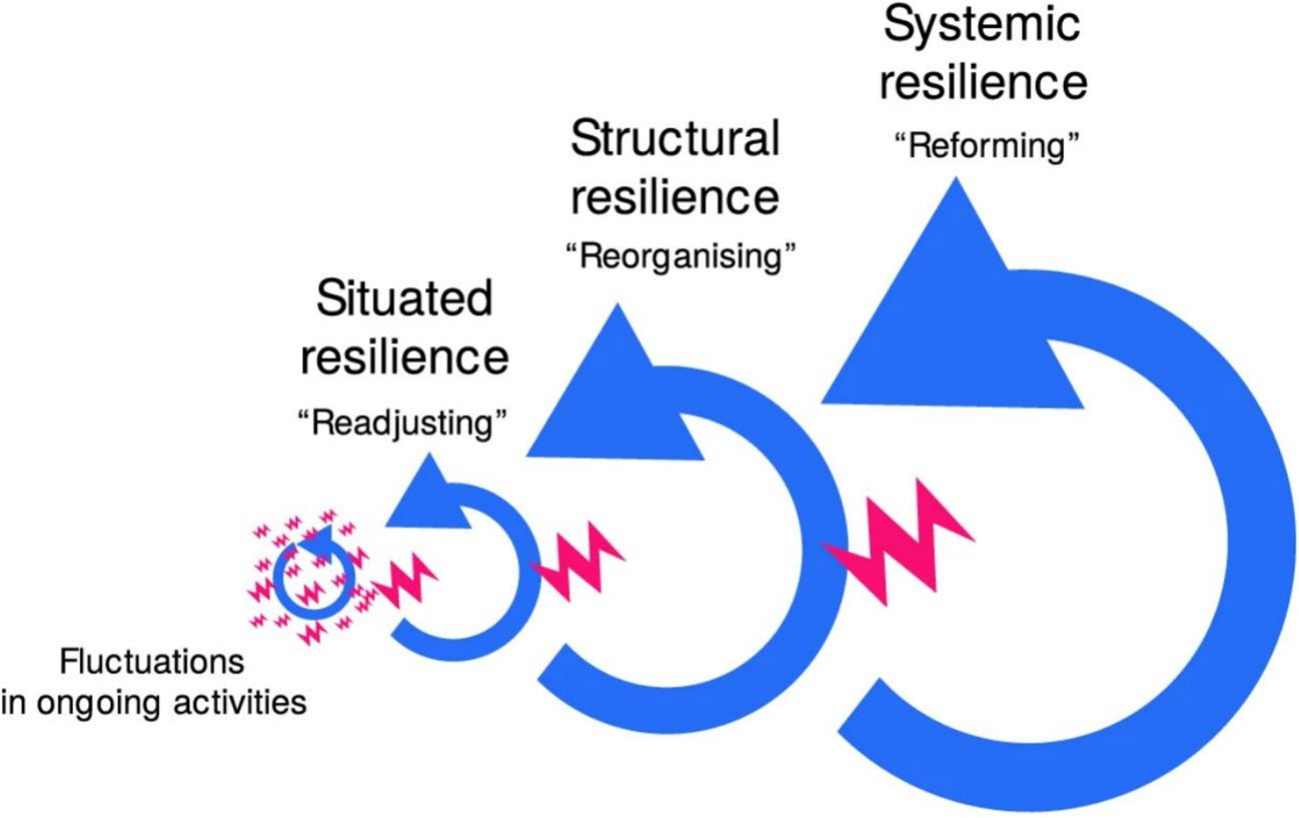
Illustrative description of Moments of Resilience theory [25]. (Reproduced under Creative Commons license)

One way of looking at resilience is to consider the temporal aspect of this phenomenon. This is provided by the Moments of Resilience theory [26] which has three levels (micro, meso, and macro) and describes how resilience can emerge and change over time (see Fig. 1). The micro level is termed ‘situated’ and refers to the immediate events around healthcare delivery. The meso level is termed ‘structural’ and involves the restructuring of socio-technical resources over a longer period of time. Finally, the macro level is termed ‘systemic’ and involves the reconfiguration of socio-technical resources occurring over a significant period of time.

Moments of Resilience Theory offers an attractive approach for conducting a qualitative framework synthesis using heterogeneous data. This is because the main concept within the theory relates to time and how resilience manifests itself over time. As time is a universal concept, the qualitative data within the existing evidence base will include descriptions of change over time despite the specific research aim or methods used within a study. Therefore, the theory should provide a framework to capture, organise and interpret this data and relate it to resilience. This will further our understanding of how individuals who carry out healthcare tasks at home avoid harm.

The aim of this systematic review is to use the Moments of Resilience theory to explore parents' and carers’ experiences of administering medicines to children at home.

## Protocol

This systematic review will be conducted in the form of a qualitative synthesis design using the framework synthesis method described by the Cochrane-Campbell Handbook for Qualitative Evidence Synthesis [27]. The final report produced will follow the Enhancing Transparency in Reporting the Synthesis of Qualitative Research (ENTREQ) [28]. This review has been registered on the International Prospective Register of Systematic Reviews database (PROSPERO) #487,154.

### Research question

The primary research question for this review is the following:

- To what extent do the experiences of parents and carers show resilience when administering medicines to children at home?

If these experiences demonstrate resilience, then further questions will be asked:

- Where and in what ways do parents and carers demonstrate resilience when administering medicines to children at home?
- What resources, processes, and adjustments do parents and carers use to improve their resilience when administering medicines to children at home?
- What influencing factors do parents and carers experience that may improve or diminish their ability to be resilient when administering medicines to children at home?

### Inclusion and exclusion criteria

The following inclusion criteria will be used:

- Empirical research in manuscript format published in peer-reviewed journals.
- Qualitative methods, or mixed methods where the qualitative data component can be clearly extracted
- Data originated from parents and carers.
- Data is related to the administration of prescribed medicines within the home.
- Data may include, but not be limited to, transcripts (e.g. from interviews or focus groups), online message boards, and diaries.
- The prescribed medicine(s) administered are to children, defined as less than 18 years of age.

The following exclusion criteria will be used:

- Quantitative data
- Data where the person administering the medicines is under 18 years of age (i.e. the self-administration of medicines)
- Data where the medicine is administered in another setting outside the home (e.g. foster home, school)
- Grey literature

### Search strategy

An iterative search strategy will be used to ensure all relevant studies are identified [29]. This approach has been used by previous qualitative reviews [30], and balances the strengths and weaknesses of the various methods of searching. For example, developing search strategies for the major databases is a time-efficient way of retrieving many studies, but may demonstrate poor precision due to the heterogeneity of keywords in the qualitative literature [31]. Therefore, to supplement this a ‘berry-picking model’ will be used which will include footnote chasing, citation searching, author searching, and hand searching [31]. Searching will stop when the research team agrees that finding any further studies is unlikely to have a significant impact on the findings of the review.

Initially, a scoping search will be conducted using the major databases Embase, PyscINFO, CINAHL, Cochrane, and PubMed. The basis for the search strategy will use the Population, Exposure, and Outcome (PEO) framework [32]. The three main search concepts are the following: parents and carers (population), administration of medicines (exposure), and safe administration in the home environment (outcome). As this review is applying a deductive method using a safety theory to identify safe outcomes, the outcome concept will be shortened to the home environment.

The search strategy, including all identified keywords and index terms, will be developed with an information specialist and adapted for each database [33] (see Table 1 for an example of a specific search strategy for the Embase database).

**Table 1:** Example search strategy

	Concept	Search strategy	Totals
#1	Parent or carer	(exp parent/OR parent$.ti,ab. OR exp legal guardian/OR exp caregiver/OR caregive*.ti,ab. OR mother$.ti,ab. OR father$.ti,ab.)	1,216,021
#2	Medicine administration	(exp prescription/OR prescription$.ti,ab. OR exp drug therapy/ OR (giv* adj4 medic*).ti,ab. OR (medic* adj4 administ*).ti,ab. OR (drug adj4 administ*).ti,ab.)	4,102,746
#3	At home	(exp primary health care/OR exp home environment/OR exp home care services/OR home*.ti,ab. OR house*.ti,ab.)	1,398,748
#4	–	#1 AND #2 AND #3	11,040

Database: Embase Classic + Embase < 1947 to 2024 March 04 >

Date of search: March 14th 2024

Total retrieved results: 11,040

The Preferred Reporting Items for Systematic Reviews and Meta-Analyses (PRISMA) will be followed for the reporting of searches [34].

### Study selection

Following the initial major database searches, all identified citations will be loaded into Rayyan® (Web application) and duplicates removed. The screening will be done in two stages. The first stage will be by title only, except if a title is ambiguous. Any ambiguous titles will have their abstract reviewed to determine the final decision.

Screeners will identify studies which are relevant to the research aim (i.e. paper which are qualitative research of parent and/or caregiver experiences of administering medicines to children at home). Two reviewers will independently check 10% of the titles and compare decisions. In the case of > 10% disagreement then review criteria will be discussed between all authors and refined. After this calibration, a single reviewer will check the remaining studies.

Studies clearing the first screen will then have their full text assessed in detail against the inclusion criteria by two independent reviewers. Reasons for exclusion of full-text studies will be recorded and reported in the systematic review. Any disagreements that arise between the two independent reviewers at each stage of the study selection process will be resolved through discussion, or with a third reviewer from the study supervision team.

### Assessment of methodological quality

Methodological quality will be assessed by using the Joanna Briggs Institute (JBI) critical appraisal checklist for qualitative research [35]. Authors of papers will be contacted to request missing or additional data for clarification, where required. All studies will be assessed independently by two reviewers. Quality assessment will be compared and any disagreements about inclusion or exclusion that arise between the reviewers will be resolved through discussion, or with a third reviewer. The results of critical appraisal will be reported in narrative form and in a table.

### Data extraction and transformation

Reviewers will extract text that relates to parent or caregiver experiences, the administration of medicines to children in the home, and disruptions or adaptions to that process that have occurred over time. Two reviewers will independently extract data using a study-specific Microsoft Excel® Spreadsheet and discuss any discrepancies between the extracted data.

The spreadsheet will be used to organise the data related to the study characteristics such as author(s), year of publication, country, aims of study, study design, length, participants, and results.

For qualitative studies (and qualitative sections of mixed methods studies), findings relevant to the review question will be extracted and supported with examples (e.g. a quotation from a participant) to preserve the context and ensure validity of the findings. Findings will be taken from the results, discussion and conclusion section of papers.

### Data synthesis and integration

The qualitative synthesis will follow the framework approach described by Ritchie and Spencer [36], and further developed by Cochrane for systematic reviews [27].

This involves five stages: familiarisation, thematic framework identification, indexing, charting, mapping and interpretation. This approach can be conducted in a non-linear fashion. Some familiarisation has occurred during the development of this research question, search strategy, and stakeholder engagement which subsequently facilitated the identification of an appropriate theoretical framework.

The Moments of Resilience theory will be used as a framework for the analysis [25]. This has been chosen as it will allow for the mapping of the data against three recognised resilience concepts: situated, structural, and systemic. Data from the extraction phase will be indexed and charted according to the framework using a Microsoft Excel® spreadsheet. The final synthesis will then be completed by mapping and interpreting these findings. Any findings which do not fit within the framework will be collated and included in the results. This will allow for further discussion and evaluation of Moments of Resilience theory and the framework synthesis approach used in this study.

## Discussion

This protocol describes a novel review to explore an important patient safety issue. The framework synthesis will provide the foundations to synthesis and build upon the existing qualitative evidence base. A strength of this review will be not only to identify, describe and collate the existing studies but also to learn about the application of Resilience Healthcare theory to a medication safety topic.

For researchers working in the area of patient safety, it is important not to forget that the ultimate aim must be to deliver improvements for patients and their families. This review will inform a series of studies that will work towards this aim, using Resilience Healthcare theory, to reform the existing support available to families, or by designing and implementing new interventions entirely.

## Data Availability

Not applicable.
